# Wild, insectivorous bats might be carriers of *Campylobacter* spp.

**DOI:** 10.1371/journal.pone.0190647

**Published:** 2018-01-11

**Authors:** Wilma C. Hazeleger, Wilma F. Jacobs-Reitsma, Peter H. C. Lina, Albert G. de Boer, Thijs Bosch, Angela H. A. M. van Hoek, Rijkelt R. Beumer

**Affiliations:** 1 Laboratory of Food Microbiology, Wageningen University & Research, Wageningen, Netherlands; 2 RIKILT Institute of Food Safety, Wageningen, Netherlands; 3 Naturalis Biodiversity Center, Leiden, Netherlands; 4 Wageningen Bioveterinary Research, Wageningen University & Research, Lelystad, Netherlands; 5 Ad Hoc Ecotechniek, Arnhem, Netherlands; 6 RIVM National Institute for Public Health and the Environment, Bilthoven, Netherlands; CSIRO, AUSTRALIA

## Abstract

**Background:**

The transmission cycles of the foodborne pathogens *Campylobacter* and *Salmonella* are not fully elucidated. Knowledge of these cycles may help reduce the transmission of these pathogens to humans.

**Methodology/principal findings:**

The presence of campylobacters and salmonellas was examined in 631 fresh fecal samples of wild insectivorous bats using a specially developed method for the simultaneous isolation of low numbers of these pathogens in small-sized fecal samples (≤ 0.1 g). *Salmonella* was not detected in the feces samples, but thermotolerant campylobacters were confirmed in 3% (n = 17) of the bats examined and these pathogens were found in six different bat species, at different sites, in different ecosystems during the whole flying season of bats. Molecular typing of the 17 isolated strains indicated *C*. *jejuni* (n = 9), *C*. *coli* (n = 7) and *C*. *lari* (n = 1), including genotypes also found in humans, wildlife, environmental samples and poultry. Six strains showed unique sequence types.

**Conclusion/significance:**

This study shows that insectivorous bats are not only carriers of viral pathogens, but they can also be relevant for the transmission of bacterial pathogens. Bats should be considered as carriers and potential transmitters of *Campylobacter* and, where possible, contact between bats (bat feces) and food or feed should be avoided.

## Introduction

*Campylobacter* and *Salmonella* are the two most important zoonotic bacteria in Europe [1], and these pathogens are commonly transmitted to humans via food, often of animal origin. Since the transmission cycles of both bacteria are not fully revealed, it is useful to search for possible reservoirs in the environment since different species of wildlife, such as wild birds, are known to be potential carriers of *Campylobacter* [2–4] and *Salmonella* [5]. So far, bats are identified to be potential carriers of mainly viral pathogens [6, 7] but they might also be relevant in the transmission cycles of *Campylobacter* and *Salmonella*. Thus far, information on the presence of *Campylobacter* in bats is rare [8] or only suspected [9], but other bacterial pathogens have been isolated occasionally from bats around the world, such as *Salmonella* and *Shigella* [10]. In Western Europe, all bat species are insectivorous (Microchiroptera). The fact that insects are able to transmit *Campylobacter* [11–13] or *Salmonella* [14] for instance via feces of farm animals or water birds, leads to the assumption that they might be a source of infection for bats as well. By contaminating water, crops, fruit, feed or soil with their feces, it could be speculated that infected bats might in turn play a part in the transmission of these bacteria. An opportunity arose to participate in an ongoing surveillance on viruses in bats. In this investigation fresh fecal samples of wild bats were examined for the presence of *Campylobacter* and *Salmonella*. For epidemiological purposes, 17 isolated *Campylobacter* strains were typed using real-time PCR, matrix-assisted laser desorption and ionization-time-of-flight mass spectrometry (MALDI-TOF MS) and multilocus sequence typing (MLST) was performed by Sanger sequencing and/or whole genome sequencing (WGS).

## Material and methods

### Ethical statement

All procedures were carried out in strict compliance with the Flora and Fauna Act licenses FF/75A/2003/150 and FF/75A/2003/169/a/b, issued by the former Dutch Ministry of Agriculture, Nature and Food Quality, and with permission of all site owners (Staatsbosbeheer; Limburgs Landschap). All bats were released within one hour at the point of capture.

### Bacterial strains

*Campylobacter jejuni* C356 and *Salmonella* Livingstone (both from the culture collection of the RIVM, Bilthoven, Netherlands) were cultured in Brain Heart Infusion broth (BHI, Becton Dickinson and Company, Sparks, USA) for use as positive controls. *Campylobacter* cultures were grown for two days at 41.5°C in micro-aerobic atmosphere achieved by flushing jars with the appropriate gas mixture (10% CO_2_, 5% O_2_ and 85% N_2_) and *Salmonella* was cultured for 24 h at 37°C, unless stated otherwise. All strains were maintained as overnight cultures in BHI with 15% glycerol at -80°C.

### Bat feces sampling

**Fig 1:**
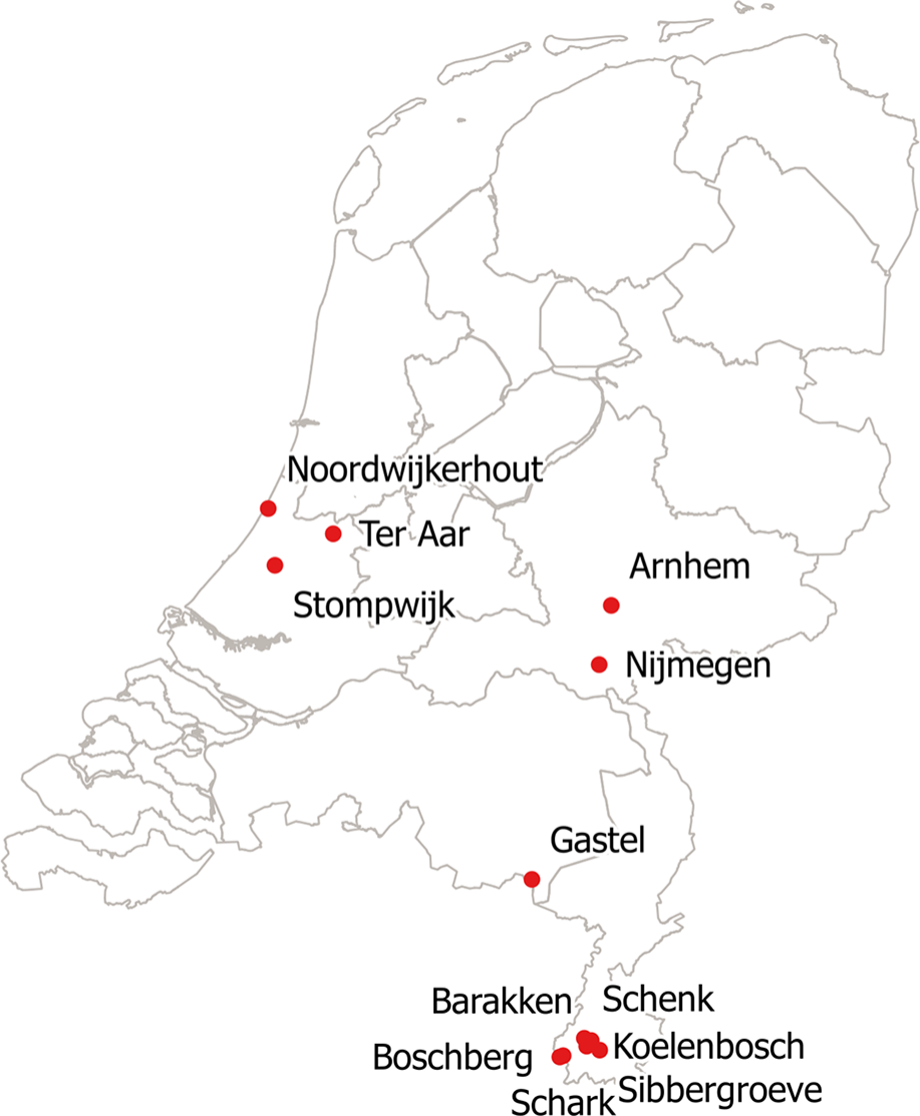
Catching sites of *Campylobacter*-positive bats in the Netherlands.

Active surveillance programs examining bats for the presence of several viruses such as rabies provided fecal samples from 631 bats for this study. In 2007 and 2008, bats were caught during the flying season (April-October) in several regions of the Netherlands (Fig 1). With exception of a few grounded bats, bats were caught with mist-nets in their foraging habitats in forests or over water bodies, and in the southernmost part of the country in the province of Limburg at swarming sites at the entrances of limestone mines [15]. After catching, each bat was kept (not sedated) in a sterile cotton bag for about 30 min for the collection of fecal pellets after which, if possible, the species, sex, age and reproductive status were determined. All bats were released at their capture site. Fecal samples were taken with swabs (Transystem Amies medium transport swabs; 108.USE, Copan Diagnostics Inc, Murietta, USA) either from the cotton bag or directly from the animals if defecation occurred during handling of the bats. Swab samples were kept in transport medium at 0–4°C for a maximum of two days before microbiological examination was started.

### Isolation of *Salmonella* and *Campylobacter*

A method was developed for simultaneous isolation of low numbers of *Salmonella* and *Campylobacter* both from one small-sized fecal sample (≤ 0.1 g). In a pre-trial, using all components in the transport swabs and -media, it was possible to easily recover both pathogens from fecal samples after two to seven days of storage of the swabs (at 0–4°C) at levels as low as 10–100 CFU per swab [16]. Methods and media described in the ISO-protocols for *Salmonella* [17] and *Campylobacter* [18] were adapted as follows: for direct isolation of *Campylobacter*, the swabs containing fecal material were streaked onto modified charcoal cefoperazone deoxycholate agar (mCCDA) plates and subsequently put in 10 ml buffered peptone water (BPW). The transport medium of the transport tube was mixed with 5 ml of Bolton Broth (BB) and incubated for 4 h at 37°C, and subsequently for 44 h at 41.5°C in micro-aerobic atmosphere (see above). After mixing the BPW suspension plus swab, one ml was transferred to 9 ml of Preston Broth (PB) for selective enrichment of *Campylobacter*, since this medium has shown to give better selectivity compared to BB [19, 20]. The contents of PB- and BB-tubes were streaked onto mCCDA after 24 and 48 h of incubation at 41.5°C in micro-aerobic conditions. The mCCDA plates were incubated under micro-aerobic conditions for 48 h at 41.5°C. Suspect colonies were confirmed by microscopy and a latex agglutination test for *Campylobacter* (M46CE, Microgen Bioproducts, Camberley UK). For detection of *Salmonella*, the remainder of the BPW was incubated at 37°C for 16–20 h after which three drops of BPW were spotted onto the center of a modified semi-solid rappaport vassiliadis (MSRV) plate (incubated at 41.5°C for 24 and 48 h). Suspect growth was then streaked onto brilliant green agar/xylose desoxycholate agar plates (BGA/XLD) which were incubated for 24 h at 37°C. Suspect colonies from BGA/XLD were streaked to neutral medium (Nutrient agar) and further confirmed for *Salmonella* using Wellcolex Colour Salmonella (Remel Europe, Dartford, UK). All media except BHI were purchased from Oxoid, Basingstoke, UK.

### Typing of *Campylobacter* strains

The isolated strains were initially tested using duplex real-time PCR as described previously [21] to determine *C*. *jejuni* and *C*. *coli*. Furthermore, MALDI-TOF MS analyses were performed to obtain further information on the remaining unidentified strains with a Bruker Daltonics MALDI Biotyper [22] using a database collection of strains as mentioned in S1 Table. Multilocus sequence typing (MLST) was done by Sanger sequencing using loci *aspA*, *glnA*, *gltA*, *glyA*, *pgm*, *uncA* and *tkt* [23, 24]. Instant, multilocus batch query (https://pubmlst.org/campylobacter/) [25] was done by using the MLST plugin from Bionumerics 6.1 (Applied Maths, Austin, USA).

The seventeen strains were then subjected to whole genome sequence (WGS) analysis. Strains were grown micro-aerobically in 10 ml Heart Infusion broth (bioTRADING, Mijdrecht, the Netherlands) with gentle shaking at 37°C for 24 h. Cell pellets were obtained by centrifugation, they were washed and dissolved in 200 μl DNA/RNA Shield (Zymo Research, Irvine, CA, USA). DNA isolation, fragmentation and library preparation for whole genome sequencing was outsourced to an independent service company (BaseClear, Leiden, the Netherlands). The isolates were sequenced on a HiSeq 2500 sequencer (BaseClear, Leiden, the Netherlands). *De novo* assembly of the WGS data was performed using Velvet [26]. *In silico* basic local alignment search tool (BLAST) analysis with the sequences of universal *Campylobacter* primers and probes [27] against the assembled genomes was performed to confirm the genus. Additionally, BLAST analysis with sequences of probes specific for *C*. *coli*, *C*. *jejuni*, *C*. *lari* and *C*. *upsaliensis* [28] was performed to determine the *Campylobacter* species *in silico*. *In silico* MLST was performed on the assemblies via the public *Campylobacter* MLST database (https://pubmlst.org/campylobacter/).

## Results and discussion

### Detection of *Campylobacter* and *Salmonella*

*Campylobacter-*confirmed strains were found in 17 out of 631 fecal samples (3%; Table 1), in six out of 14 different bat species from diverse habitats (Fig 1 and Table 2) throughout the flying season. These findings are in accordance with the study of Hatta et al. [29], who found partial genomes of *C*. *jejuni* and *C*. *coli* in rectal swab samples of fruit bats. However, Adesiyun et al. [30] tested gastrointestinal tracts of 377 bats in Trinidad and Tobago and did not find any campylobacters. This could be explained by climate differences or by the fact that rather than with enrichment broths, the study was carried out using selective plates, which do not easily allow growth of sub-lethally damaged cells. A quantitative metagenomic analysis of bat fecal bacteria in Finland did not show any genomic DNA of *Campylobacter*, but in this case, only one Daubenton’s Bat (*Myotis daubentonii*) was examined [31].

**Table 1:** Number of *Campylobacter-*positive bats (total number of bats sampled) in 2007 and 2008.

Bat species	2007	2008
*Eptesicus serotinus*	ns^a^	0 (29)
*Myotis bechsteinii*	ns	2 (28)
*Myotis brandtii*	ns	0 (11)
*Myotis dasycneme*	2 (25)	0 (13)
*Myotis daubentonii*	0 (15)	8 (164)
*Myotis emarginatus*	ns	1 (70)
*Myotis myotis*	ns	0 (6)
*Myotis mystacinus*	ns	0 (20)
*Myotis nattereri*	ns	0 (27)
*Nyctalus noctula*	2 (11)	0 (2)
*Pipistrellus nathusii*	0 (5)	0 (4)
*Pipistrellus pipistrellus*	0 (6)	2 (96)
*Plecotus auritus*	0 (2)	0 (24)
*Plecotus austriacus*	ns	0 (2)
Microchiroptera, not further specified	ns	0 (71)

^a^ ns: not sampled

**Table 2:** Information of isolated strains: Catching date and -location of the bats, bat species, *Campylobacter* species, Sequence Type (ST) and clonal complex (if existing).

Strain	Date	Location^a^	Bat species	*Campylobacter* species^c^	ST	Clonal complex
1	04-18-07	Stompwijk (ZH)	*Myotis dasycneme*	*jejuni*	991	ST-692
2	06-08-07	Noordwijkerhout (ZH)	*Nyctalus noctula*	*jejuni*	432	ST-61
3	06-08-07	Noordwijkerhout (ZH)	*Nyctalus noctula*	*jejuni*	583	ST-45
4	06-19-07	Ter Aar (ZH)	*Myotis dasycneme*	*jejuni*	704	-
5	07-04-08	Gastel (NB)	*Pipistrellus pipistrellus*	*jejuni*	334	ST-45
6	07-15-08	Arnhem/Nijmegen area (GLD)	*Myotis daubentonii*	*coli*	2007	-
7	07-15-08	Arnhem/Nijmegen area (GLD)	*Myotis daubentonii*	*coli*	2007	-
8	07-15-08	Arnhem/Nijmegen area (GLD)	*Myotis daubentonii*	*coli*	9007^d^	-
9	07-15-08	Arnhem/Nijmegen area (GLD)	*Myotis daubentonii*	*coli*	8159^d^	-
10	08-04-08	Schark^b^ (L)	*Pipistrellus pipistrellus*	*jejuni*	19	ST-21
11	08-18-08	Schenk^b^ (L)	*Myotis daubentonii*	*coli*	7255^d^	-
12	08-25-08	Barakken^b^ (L)	*Myotis daubentonii*	*lari*	138^d^	-
13	08-25-08	Koelenbosch^b^ (L)	*Myotis daubentonii*	*coli*	9005^d^	-
14	09-01-08	Boschberg^b^ (L)	*Myotis bechsteinii*	*jejuni*	267	ST-283
15	09-08-08	Boschberg^b^ (L)	*Myotis daubentonii*	*coli*	9006^d^	-
16	09-15-08	Sibbergroeve^b^ (L)	*Myotis emarginatus*	*jejuni*	48	ST-48
17	09-22-08	Boschberg^b^ (L)	*Myotis bechsteinii*	*jejuni*	2274	-

^a^ ZH = province of Southern Holland; NB = province of Northern Brabant; GLD = province of Gelderland; L = province of Limburg

^b^ Limestone mine

^c^ For technical details about species identification and typing is referred to S3 Table

^d^ New MLST registered

From the fecal samples of the bats, multiple routes were followed to maximize the chance of *Campylobacter* isolation; direct streak on mCCDA, or after enrichment in BB and PB. From two *Campylobacter*-positive samples, bacterial strains were isolated via all routes (S2 Table). However, in 9 out of the 17 samples (53%), the bacterium was only isolated via the PB route. In most of those cases, the plates from the BB enrichment were overgrown with contaminating flora, preventing recognition and isolation of *Campylobacter* colonies. This confirms other findings of PB being more selective than BB in the detection of *Campylobacter* [19, 20, 32]. This study was biased with respect to catching sites due to dependency on ongoing research, which was mainly focusing on bats in the middle and southern part of the Netherlands. No correlation could be found between gender of the bats and *Campylobacter* carriage. Except for the two *Campylobacter*-positive Noctule Bats (*Nyctalus noctula*) that had diarrhea, bats generally looked healthy with solid droppings, indicating that most bats are probably healthy carriers. *Salmonella* was not isolated from any of the samples in the present study. Since the aim was to determine the presence of both *Campylobacter* and *Salmonella*, splitting-up the material and the small sample quantity (<10–100 mg) could lead to an underestimation of the number of positive animals and this could also explain the fact that *Salmonella* was not found. Furthermore, it has been reported that *Salmonella* shedding in animals like chickens [13] and pigs [33] can be intermittent; this could also be the case in bats. Other studies did mention presence of *Salmonella* in vespertilionid bats, for instance in 0.6% of 486 carcasses of deceased animals in Germany [34] or in 2% of 96 live bats in the Philippines, but in the latter case, *Salmonella* could not be cultured and was found only with PCR techniques [35]. *Salmonella* spp. have also been occasionally isolated from other bat families [30,36,37].

### Genetic characterization

*C*. *jejuni* was the most common species found (9 times; Table 2). Identification to the species level proved to be difficult for seven strains. Six strains were negative in the real-time PCR for *C*. *jejuni* or *C*. *coli* but using MALDI-TOF MS, these strains were designated as probably *Campylobacter*, with unreliable *C*. *coli* indication (Table 2, S3 Table). One strain (strain 12) could not be further identified with either of these techniques. Fortunately, WGS data analysis did allow speciation of all strains. *In silico* basic local alignment search tool (BLAST) analysis with the sequences of universal *Campylobacter* primers and probes [27] against the assembled genome of strain 12 showed 100% matches, confirming that it was a *Campylobacter* (data not shown). Additionally, BLAST analysis with sequences of probes specific for *C*. *coli*, *C*. *jejuni*, *C*. *lari* and *C*. *upsaliensis* [28], revealed the highest match of 87% with *C*. *lari* (Table 2 and S3 Table). Because of this relatively low similarity, strain 12 was also typed with the SpeciesFinder 1.2 service at the Center for Genomic Epidemiology website (https://cge.cbs.dtu.dk/services/SpeciesFinder/). The outcome was again *C*. *lari* (data not shown). For six isolates new alleles and STs were assigned by the curators of the *jejuni/coli* and non-*jejuni/coli* MLST databases (S3 Table).

MLST results indicated that *Campylobacter* strains isolated from bats were similar to the types previously found in various sources such as humans, environmental waters, food, poultry and other animals (Table 3 and S3 Table; [27]). The sources of infection for bats are most probably other bats in the same colony, contaminated water or insects that were in contact with contaminated water or animal feces. A total of 16 different sequence types (ST) were identified (Table 2) of which seven belonged to a clonal complex. The six different clonal complexes identified were ST-21, ST-45, ST-48, ST-61, ST-283 and ST-692 (Tables 2 and 3). Two strains within the clonal complex ST-45 were found on different dates at different locations in different bat species. Clonal complexes ST-45 and ST-61 are among the most frequently isolated genotypes in humans [38] and are also found in other studies in wildlife and water samples [39]. Six new STs were identified in six strains (Table 2 and S3 Table).

**Table 3:** Clonal complexes of *Campylobacter* sequence types found in feces from bats and common sources of isolates within the MLST database (https://pubmlst.org/campylobacter/).

Strain	Clonal complex		Number of STs in clonal complex^a^	Common sources
1		ST-692	75	Cattle, environmental waters, human, poultry, wild bird
2		ST-61	178	Cattle, environmental waters, farm environment, human, other animal, poultry, sheep
3		ST-45	345	Cat, cattle, dog, environmental waters, farm environment, human, other animal, other food, poultry, sheep, wild bird
5		ST-45	345	Cat, cattle, dog, environmental waters, farm environment, human, other animal, other food, poultry, sheep, wild bird
10		ST-21	753	Cattle, dog, environmental waters, farm environment, human, other animal, other food, pig, poultry, sheep, turkey, wild bird
14		ST-283	56	Cattle, dog, environmental waters, human, other animal, other food, potable/drinking water, poultry, sheep, wild bird
16		ST-48	220	Cattle, dog, environmental waters, farm environment, human, other animal, other food, pig, poultry, sand (bathing beach), sheep

^a^ As of Sep. 15^th^ 2017

All isolates of one bat, obtained from the different isolation methods resulted in the same MLST types. The feces samples from both Noctule Bats, sampled at the same spot and day, were positive for *Campylobacter*. However STs and clonal complexes (strains 2 and 3 in Table 2) were different, demonstrating that within local populations different *Campylobacter* types exist.

## Conclusions

In conclusion, despite the drawbacks of the methods, *Campylobacter* was found in fecal samples of six different bat species, at different sites, in different ecosystems during the whole flying season of bats. Molecular typing of the strains indicated genotypes also found in humans, wildlife, environmental samples and poultry. Therefore, bats could be considered as possible carriers and transmitters of *Campylobacter* like birds and rodents. Where possible, contact between bats (bat feces) and food or feed should be avoided.

## Supporting information

S1 Table Relevant strains present in MALDI TOF MS database used for typing of the *Campylobacter* strains. (XLSX)

S2 Table Isolation routes of *Campylobacter* from bat fecal samples. mCCDA: direct isolation on mCCDA plates; BB 24 h and BB 48 h: isolation via enrichment procedure in Bolton Broth incubated for 24 h and 48 h respectively; PB 24 h and PB 48 h: isolation via enrichment procedure in Preston Broth incubated for 24 h and 48 h respectively.
(DOCX)

S3 Table Combined confirmation- and typing data of 17 *Campylobacter* strains isolated from bats. The following techniques were used: duplex real-time PCR for determination of *C*. *jejuni* and *C*. *coli*; Multilocus Sequence typing (resulting in Sequence Types (ST)); MALDI TOF MS (Maldi); Whole Genome sequencing (WGS); *in silico* Sequence Typing (*in silico* ST). For strains 8, 16 and 17, multiple isolates were typed.
(XLSX)
