# Supplementary material for: Wild, insectivorous bats might be carriers of Campylobacter spp
Source: PLoS One. 2018 Jan 11;13(1):e0190647. doi: 10.1371/journal.pone.0190647 (PMC5764278; doi:10.1371/journal.pone.0190647)
Supplement: S2 Table — mCCDA: direct isolation on mCCDA plates; BB 24 h and BB 48 h: isolation via enrichment procedure in Bolton Broth incubated for 24 h and 48 h respectively; PB 24 h and PB 48 h: isolation via enrichment procedure in Preston Broth incubated for 24 h and 48 h respectively. (DOCX) [file pone.0190647.s002.docx]

**S2 Table. Isolation routes of *Campylobacter* from bat fecal samples.** mCCDA: direct isolation on mCCDA plates; BB 24 h and BB 48 h: isolation via enrichment procedure in Bolton Broth incubated for 24 h and 48 h respectively; PB 24 h and PB 48 h: isolation via enrichment procedure in Preston Broth incubated for 24 h and 48 h respectively.

|  | *Campylobacter* isolated via: | | | | |
| --- | --- | --- | --- | --- | --- |
| *Campylobacter* strain | mCCDA | BB 24 h | PB 24 h | BB 48 h | PB 48 h |
| 1 | + | - | - | + | + |
| 2 | + | - | + | + | + |
| 3 | - | - | + | - | - |
| 4 | + | - | + | - | + |
| 5 | - | - | + | - | + |
| 6 | + | - | + | - | + |
| 7 | - | - | + | - | + |
| 8 | - | + | + | + | + |
| 9 | - | - | + | - | + |
| 10 | - | + | + | - | + |
| 11 | - | - | + | - | + |
| 12 | + | - | + | - | - |
| 13 | - | - | + | - | + |
| 14 | - | - | + | - | + |
| 15 | - | - | + | - | + |
| 16 | - | - | + | - | + |
| 17 | + | - | - | - | - |
